# TF-343 Alleviates Diesel Exhaust Particulate-Induced Lung Inflammation via Modulation of Nuclear Factor-*κ*B Signaling

**DOI:** 10.1155/2019/8315845

**Published:** 2019-10-30

**Authors:** Dong Im Kim, Mi-Kyung Song, Seon-Hee Kim, Chan Young Park, Kyuhong Lee

**Affiliations:** ^1^National Center for Efficacy Evaluation of Respiratory Disease Products, Jeonbuk Department of Inhalation Research, Korea Institute of Toxicology, 30 Baehak1-gil, Jeongeup, Jeollabuk-do 56212, Republic of Korea; ^2^Department of Human and Environmental Toxicology, University of Science & Technology, Daejeon 34113, Republic of Korea; ^3^Sungkyun Biotech Co. Ltd., Suwon, Gyeonggi-do 164, Republic of Korea

## Abstract

Inhalation of diesel exhaust particulate (DEP) causes oxidative stress-induced lung inflammation. This study investigated the protective effects of TF-343, an antioxidant and anti-inflammatory agent, in mouse and cellular models of DEP-induced lung inflammation as well as the underlying molecular mechanisms. Mice were intratracheally instilled with DEP or vehicle (0.05% Tween 80 in saline). TF-343 was orally administered for 3 weeks. Cell counts and histological analysis of lung tissue showed that DEP exposure increased the infiltration of neutrophils and macrophages in the peribronchial/perivascular/interstitial regions, with macrophages harboring black pigments observed in alveoli. TF-343 pretreatment reduced lung inflammation caused by DEP exposure. In an in vitro study using alveolar macrophages (AMs), DEP exposure reduced cell viability and increased the levels of intracellular reactive oxygen species and inflammatory genes (IL-1*β*, inhibitor of nuclear factor- (NF-) *κ*B (I*κ*B), and Toll-like receptor 4), effects that were reduced by TF-343. A western blot analysis showed that the I*κ*B degradation-induced increase in NF-*κ*B nuclear localization caused by DEP was reversed by TF-343. In conclusion, TF-343 reduces DEP-induced lung inflammation by suppressing NF-*κ*B signaling and may protect against adverse respiratory effects caused by DEP exposure.

## 1. Introduction

Air pollution, which is made up of gases and particulate matter (PM), is a serious problem worldwide due to its adverse effects on human health. PM is a complex mixture of solid and/or liquid organic and inorganic substances suspended in the atmosphere and comprises many different types of particle depending on the emission source (e.g., natural or industrial), including diesel exhaust particles (DEP), residual oil fly ash, and urban air particles [1]. Epidemiological and toxicological studies have shown that exposure to PM can induce and exacerbate clinical manifestations of lung [2], liver [3], kidney [4], and heart [5] diseases. In particular, PM with an aerodynamic diameter < 2.5 *μ*m (i.e., PM2.5 or fine particles) can affect the human respiratory and circulatory systems, since small particles can travel deep into the respiratory tract, carrying toxic compounds into lung alveoli and blood vessels or capillaries [6, 7]. Repeated exposure to PM increases the risk of cardiovascular and respiratory diseases [8, 9].

DEP is a major PM2.5 emitted by motor vehicles and includes polycyclic aromatic hydrocarbons (PAHs), nitro-PAHs, oxygenated derivatives of PAH (ketones, quinones, and diones), heterocyclic compounds, aldehydes, and aliphatic hydrocarbons [10, 11]. In animal and human research studies, exposure to DEP has been shown to cause lung inflammation, oxidative stress, lung cancer, and cardiovascular diseases [2, 5, 8, 9]. Since 2013, diesel engine exhaust has been classified as a group 1 carcinogen by the International Agency for Research on Cancer of the World Health Organization [12]. To reduce diesel emissions from transport and other engines used in various industries, governments have used national campaigns to promote the use of personal protective equipment such as masks. However, with the rapid global industrialization, population growth, and increases in vehicular traffic, the health risk to humans is increasing in urban areas. Therefore, a considerable amount of attention has been directed towards identifying natural compounds that protect against these toxic substances. Various herbal extracts have medicinal properties such as antioxidant and anti-inflammatory activities against acute and chronic diseases including Alzheimer's or other neurodegenerative diseases and inflammation-related conditions [13]; many natural compounds have been shown to block nuclear factor- (NF-) *κ*B signaling, which plays an important role in the production of various proinflammatory mediators [14].

TF-343 is a natural mixture of oriental herbs including *Lonicera japonica*, *Adenophora triphylla* var. japonica Hara, *Taraxacum platycarpum*, *Saururus chinensis*, *Ulmus davidiana* var. japonica, *Cassia tora*, *Glycine max* Merrill, and *Glycyrrhiza uralensis* Fischer. Each component of TF-343 has demonstrated pharmacological activity, including antioxidant and anti-inflammatory effects in allergic dermatitis and detoxification of accumulated dioxin [15–32].

Given that DEP-induced lung inflammation is related to oxidative stress and inflammatory response, we investigated whether TF-343 has a protective effect in a mouse model of DEP-induced lung inflammation and the underlying mechanism of action. Our results provide evidence for the potential application of TF-343 in reducing DEP-induced lung inflammation.

## 2. Materials and Methods

### 2.1. Animals

Female Balb/c mice (Orient Bio, Seongnam, Korea) weighing 16.3 ± 0.67 g were housed in a temperature-controlled room (22°C ± 3°C) on a 12 : 12 h light/dark cycle with free access to standard laboratory chow and tap water. The mice were used for experiments after 5 days of acclimation, during which time they showed no adverse clinical signs and normal weight gain. The experiments were performed in accordance with protocols approved by the Institutional Animal Care and Use Committee of the Korea Institute of Toxicology (no. 1805-0182). The mice were randomly divided into six weight-matched experimental groups (*n* = 5 each) using the Pristima v.7.3 preclinical software program (Xybion Medical Systems Corporation, Morris Plains, NJ, USA) that were treated as follows: vehicle (VEH) control, 0.05% Tween 80-exposed mice administered distilled water; DEP+VEH, DEP-exposed mice administered distilled water; DEP+TF-343 (200), DEP-exposed mice administered 200 mg/kg TF-343; DEP+TF-343 (400), DEP-exposed mice administered 400 mg/kg TF-343; DEP+TF-343 (800), DEP-exposed mice administered 800 mg/kg TF-343; and DEP+Dexa (1), DEP-exposed mice administered 1 mg/kg dexamethasone (Dexa). At 48 h after lung exposure to vehicle or DEP, cells in bronchoalveolar lavage fluid (BALF) were analyzed and histological assessment of lung tissue was performed.

### 2.2. DEP Instillation

Mice were intratracheally instilled with 100 *μ*g DEP (SRM 2975; National Institute of Standards and Technology, Gaithersburg, MD, USA) dispersed in 50 *μ*l saline containing 0.05% Tween 80 (Sigma-Aldrich, St. Louis, MO, USA) on days 15, 18, and 21 after the first administration of TF-343 [33].

### 2.3. TF-343 and Dexa Treatment

**Figure 1:**
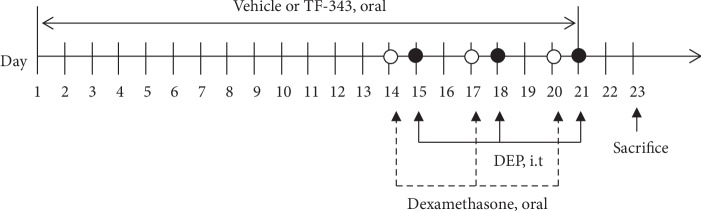
Diagram of in vivo experimental protocol. DEP: diesel exhaust particulate; i.t: intratracheal injection.

TF-343 is herbal extract from 8 medicinal plants including dandelion (*Taraxacum platycarpum*), silverwoolee (*Saururus chinensis* (*Lour.*) *Baill.*), honeysuckle (*Lonicera japonica*), ladybell (*Adenophora triphylla* var. *japonica HARA.*), sicklepod seed (*Cassia Tora*), black soybeans (*Glycine max Merrill*), bark of elm tree (*Ulmus davidiana* var. *japonica*), and liquorice (*Glycyrrhiza uralensis Fischer*). The herbs, which were purchased from Kyungdong Market (Seoul, Korea), were mixed at the same weight portions except for the half-weighed portions of black soybeans, bark of elm tree, and liquorice. Mixed herbs were extracted with hot water in a 100 l extractor (TAEIN F&C Co., Incheon, Korea). The filtered extract was powdered using a spray dryer by Sungkyun Biotech Co. Ltd. (Suwon, Korea). The powdered TF-343 was packed and stored at room temperature. Mice were orally administered TF-343 (200, 400, and 800 mg/kg body weight/day) for 3 weeks as shown in Figure 1. The amount of human intake is calculated by assigning the animal's (mouse) conversion factor of 0.08 at the 200 mg/kg/day, which is the lowest effective dose of TF-343 in the mouse lung injury model. The value can be multiplied by an adult's average weight of 60 kg, resulting in the average intake amount of 960 mg/60 kg/day. Considering that the yield of the extract solids is about 15~20% of the raw material, it can be converted into 4.8~6.4 g per day for general adults as a dry raw material. All of the herbal medicines that constitute TF-343 are food grade certified by the Ministry of Food and Drug Safety (MFDS) and can be safely consumed without restriction on the daily intake. Dexa (1 mg/kg body weight/day) as positive control was orally administered on days 14, 17, and 20.

### 2.4. BALF Preparation

At 48 h after the last DEP instillation, mice were anesthetized with isoflurane and exsanguinated. The left lung was ligated, and the right lungs were gently lavaged three times via the tracheal tube with a total volume of 0.7 ml PBS. The total number of cells in the collected BALF was counted with a NucleoCounter (NC-250; ChemoMetec, Gydevang, Denmark). For counts of different cell types, BALF cell smears were prepared using Cytospin (Thermo Fisher Scientific, Waltham, MA, USA) and stained with Diff-Quik solution (Dade Diagnostics, Aguada, Puerto Rico). A total of 200 cells per slide were counted.

### 2.5. Histological Analysis

On day 23 after the first administration of TF-343, mice were sacrificed for histological analysis. Lung tissue was removed and fixed in 10% (*v*/*v*) neutral-buffered formalin and then dehydrated, embedded in paraffin, and cut into 4 *μ*m sections that were deparaffinized with xylene and then stained with hematoxylin and eosin (H&E; Sigma-Aldrich). Stained sections were analyzed under a light microscope (Axio Imager M1; Carl Zeiss, Oberkochen, Germany). The degree of inflammation was scored on a scale of 0 to 4 as previously described [34].

### 2.6. Culture of Murine Alveolar Macrophages (AMs) and TF-343 Treatment

The MH-S murine AM cell line (CRL-2019) was purchased from the American Type Culture Collection (Manassas, VA, USA) and maintained in a Roswell Park Memorial Institute (RPMI) 1640 medium (Gibco, Grand Island, NY, USA) supplemented with 10% fetal bovine serum and 1% penicillin-streptomycin solution (Gibco). The cells were cultured at 37°C in a humidified atmosphere of 5% CO_2_. Cells were pretreated with TF-343 (100 or 200 *μ*g/ml) overnight before adding DEP (100 *μ*g/ml); 3 or 48 h later, the cells were washed twice with PBS and harvested.

### 2.7. Cell Viability Assay

To assess the toxicity of TF-343, we evaluated cell viability with the 3-(4,5 dimethylthiazol-2-thiazyl)-2,5-diphenyl-tetrazolium bromide (MTT; Sigma-Aldrich) assay. At 3 h after DEP stimulation, MTT solution (1 mg/ml) was added to the cells, followed by incubation at 37°C for 3 h. The supernatant was removed and the formazan crystals were dissolved in 100 *μ*l dimethyl sulfoxide (Junsei Chemical Co., Tokyo, Japan). Absorbance was measured at 570 nm and the background control at 690 nm with a microplate reader (BioTek, Winooski, VT, USA).

### 2.8. Measurement of Reactive Oxygen Species (ROS) Level

AMs were incubated for 30 min at 37°C with a serum-free RPMI 1640 medium containing 3.3 *μ*mol/l 2′,7′-dichlorofluorescein diacetate (DCF-DA) (Thermo Fisher Scientific) for ROS detection. The cells were fixed with 3.7% formalin in a complete medium for 15 min at 37°C and washed with PBS (Gibco). DCF-DA intensity in the cells was immediately measured at 495 nm (excitation)/529 nm (emission) with a microplate reader. ROS production in the cells is represented as a percentage of DCF-DA intensity relative to cell viability in each well, which was defined as 100%.

### 2.9. RNA Extraction and Quantitative Real-Time PCR

RNA was extracted from AMs using the RNeasy Mini kit (Qiagen, Venlo, Netherlands) according to the manufacturer's protocol and was quantified by measuring the absorption at 260 nm; 1 *μ*g was then reverse transcribed to cDNA using the ImProm-II reverse transcription system (Promega, Madison, WI, USA). The cDNA was amplified with the QuantStudio5 real-time PCR system using the SYBR Green PCR Master Mix (Applied Biosystems, Foster City, CA, USA) and the following sense and antisense primers: IL-1*β*, 5′-CAACCAACAAGTGATATTCTCCATG-3′ and 5′-ATCCACACTCTCCAGCTGCA-3′; Toll-like receptor (TLR), 4 5′-AAACGGCAACTTGGACCTGA-3′ and 5′-AGCTTAGCAG CCATGTGTTCCA-3′; inhibitor of NF-*κ*B (I*κ*B), 5′-CTACACCTTGCCTGTGAGCA-3′ and 5′-TCCTGAGCATTGACATCAGC-3′; and actin, 5′-GGCACCACACCTTCTACAATG-3′ and 5′-GGGGTGTTGAAGGTCTCAAAC-3′. Briefly, the 20 *μ*l reaction mixtures contained 10 *μ*l SYBR Green PCR Master Mix (Applied Biosystems), 2.5 pmoles of each primer, and 0.5 *μ*l nondiluted first-strand cDNA. The cycling conditions were as follows: 95°C for 10 min, followed by 40 cycles of 95°C for 15 s and 59°C for 1 min. The cycle threshold method was used to calculate relative changes in target gene expression using QuantStudio Design and Analysis v.1.4 software (Applied Biosystems). Target gene expression levels were normalized to that of actin and are expressed as fold change. The normalized value of the target gene expression level in the control group was set to 1.

### 2.10. Preparation of Cytosolic and Nuclear Protein Extracts and Western Blot Analysis

Cells were homogenized in NE-PER Nuclear and Cytoplasmic Extraction Reagent (Thermo Fisher Scientific) according to the manufacturer's protocols, and protein concentrations were determined using the Bradford reagent (Bio-Rad, Hercules, CA, USA). Proteins were separated by SDS-PAGE at 120 V for 90 min, then transferred to a polyvinylidene difluoride membrane (Bio-Rad) at 250 mA for 90 min by wet transfer. Nonspecific binding was blocked by incubating the membrane in 5% nonfat dry milk in Tris-buffered saline with Tween 20 (25 mmol/l Tris (pH 7.5), 150 mmol/l NaCl, and 0.1% Tween 20) for 1 h, followed by overnight incubation at 4°C with antibodies against NF-*κ*B p65, I*κ*B, and lamin B (all from Abcam, Cambridge, MA) and actin (Santa Cruz Biotechnology, Santa Cruz, CA, USA). Horseradish peroxidase-conjugated anti-rabbit or anti-mouse IgG (Cell Signaling Technology, Beverly, MA, USA) was used to detect antibody binding, which was visualized using the ChemiDoc MP imaging system (Bio-Rad) after treatment with enhanced chemiluminescence reagent (Thermo Fisher Scientific). Densitometric analysis of each band was carried out using Image Lab software (Bio-Rad). For quantification of specific bands, a square of the same size was drawn around each band for density measurement, and the value was adjusted to the background density near that band. The results are expressed as a relative ratio of the target to the reference protein, with the relative ratio of the target protein of the control group set to 1.

### 2.11. Statistical Analysis

Statistical analyses were performed using SigmaPlot v.12 software (Systat, San Jose, CA, USA). Data are expressed as mean ± SD. The data were tested for normality with the Shapiro-Wilk test. Statistical comparisons were performed by one-way analysis of variance followed by the Tukey or Dunnett test. Comparisons between two groups were carried out with the *t* test for paired variables or with the Mann-Whitney *U* test for unpaired variables. *P* < 0.05 was considered statistically significant.

## 3. Results

### 3.1. Changes in Body and Organ Weights

**Figure 2:**
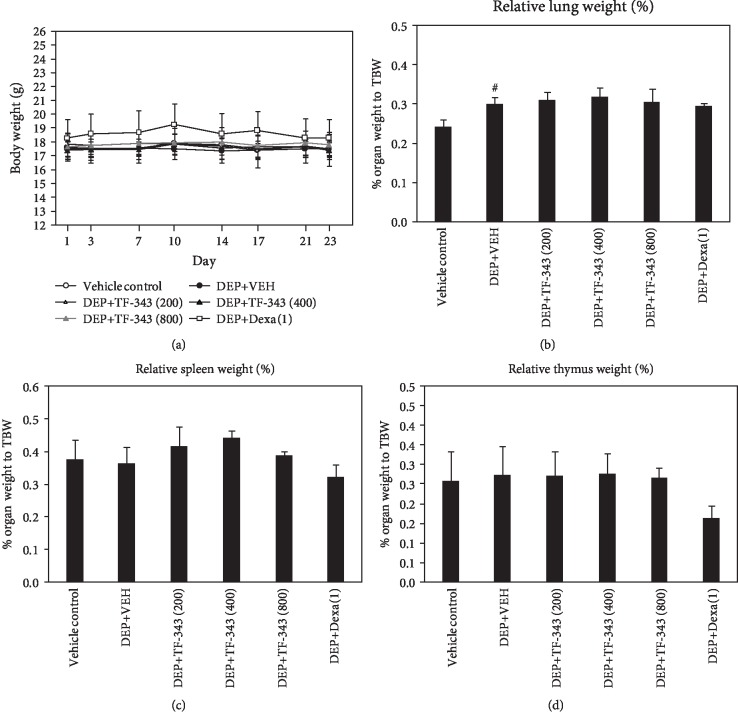
Changes in (a) body weight and (b-d) relative organ weight in DEP- and vehicle-treated mice. Lung (b), spleen (c), and thymus (d) weights were calculated with the following formula: relative organ weight = organ weight (g)/final body weight (g) × 100%. Data represent mean ± SD (*n* = 5 per group). ^#^*P* < 0.05 vs. vehicle control.

Lung injury was induced in mice by DEP instillation (see Figure 1). Body weight remained constant during the experimental period, and no differences were observed among groups (see Figure 2(a)). Relative weight of the lung was significantly increased in DEP-exposed mice as compared to vehicle control mice (see Figure 2(b)). There were no differences in the relative weights of the spleen (see Figure 2(c)) and thymus (see Figure 2(d)) in DEP-exposed as compared to vehicle control mice.

### 3.2. TF-343 Alleviates DEP-Induced Lung Inflammation in Mice

**Figure 3:**
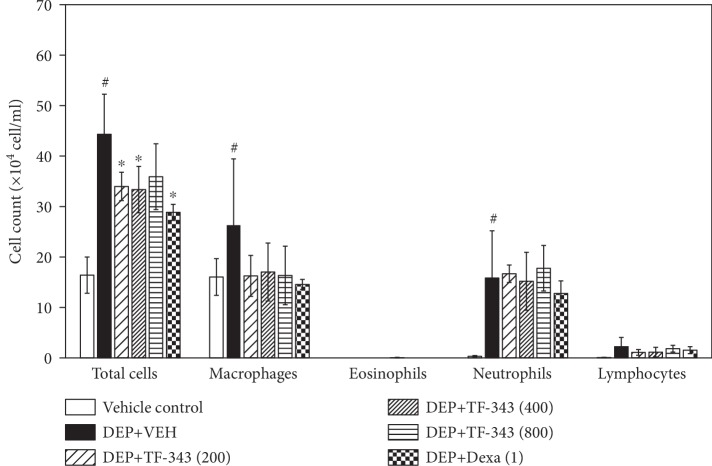
Changes in the cell population of BALF from mice treated with vehicle (VEH) or DEP, alone or in conjunction with TF-343 (200, 400, or 800 mg/kg) or Dexa (1 mg/kg). Data represent mean ± SD (*n* = 5 per group). ^#^*P* < 0.05 vs. vehicle control, ^∗^*P* < 0.05 vs. DEP+VEH.

**Figure 4:**
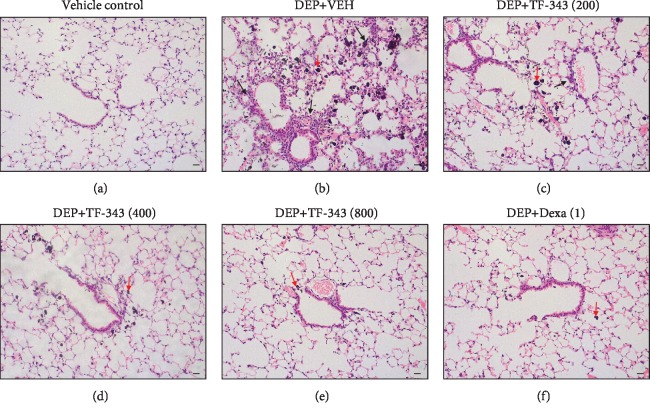
Histological changes in lung tissue caused by DEP treatment. Representative H&E-stained sections of the lung obtained from mice treated with (a) vehicle or (b-f) DEP alone (b) or in conjunction with TF-343 at 200 mg/kg (c), 400 mg/kg (d), or 800 mg/kg (e) or Dexa (1 mg/kg) (f). Red and black arrows indicate black particle-laden alveolar macrophages and inflammatory cells, respectively. Scale bars, 20 *μ*m.

To determine whether TF-343 reduces lung inflammation, we analyzed the number of inflammatory cells in BALF and histological changes in lung tissue following DEP exposure. Total cells and the number of inflammatory cells including macrophages and neutrophils in BALF were increased in the DEP-treated as compared to the vehicle control group (see Figure 3). H&E staining of lung tissue sections revealed that DEP exposure resulted in the accumulation of black particle-laden AMs (red arrows), with residual black particles detected in alveoli; this was accompanied by inflammatory cell infiltration (black arrows) in the peribronchiolar, perivascular, and interstitial regions (see Figure 4). Pretreatment with TF-343 and Dexa reduced DEP-induced inflammatory cell infiltration in BALF (see Figure 3) and lung tissues (see Figure 4) relative to mice treated with vehicle. This was confirmed by histological scoring of infiltrated inflammatory cells and AMs (see Table 1).

### 3.3. Effect of TF-343 on Oxidative Stress and Cytotoxicity in DEP-Stimulated AMs

**Figure 5:**
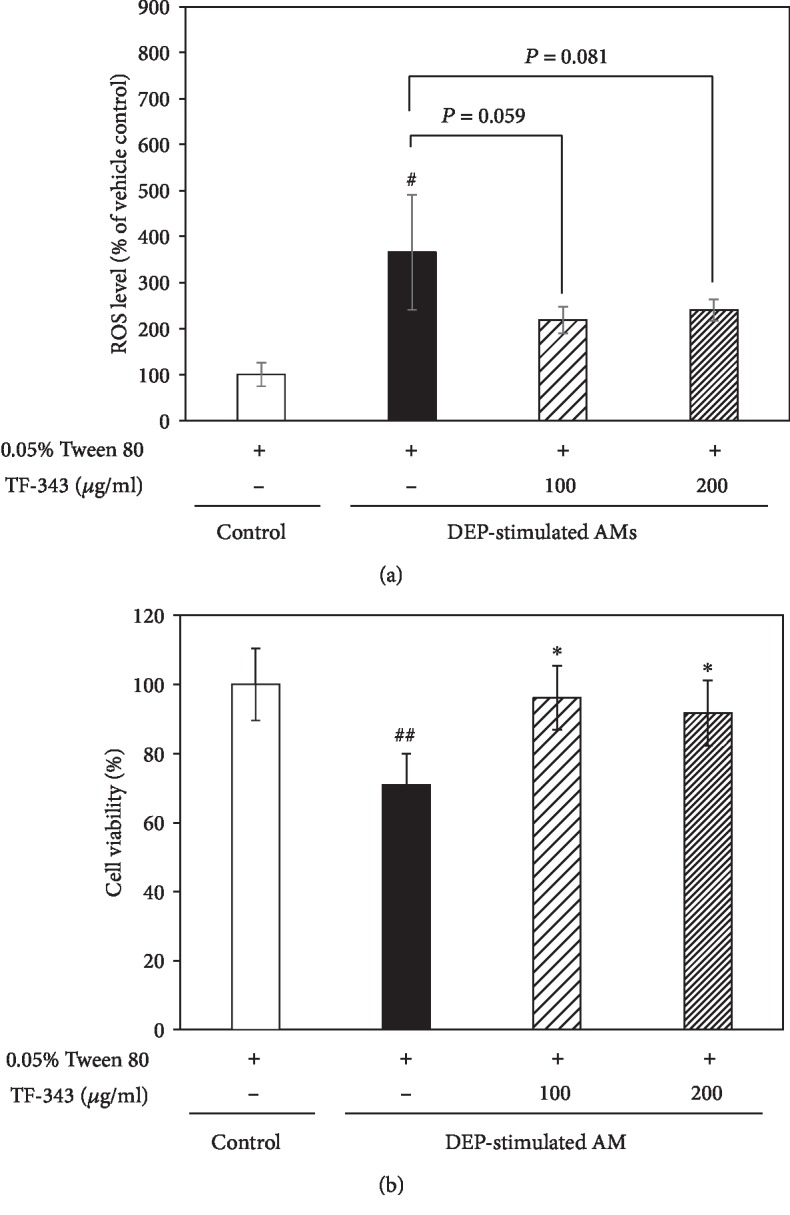
Intracellular ROS levels and viability of DEP-treated AMs. (a) ROS levels were measured by DCF-DA staining. Data represent mean ± SD (*n* = 3 per group). ^#^*P* < 0.05 vs. control. (b) Evaluation of cytotoxicity with the MTT assay. Data represent mean ± SD (*n* = 8 per group). ^##^*P* < 0.01 vs. control, ^∗^*P* < 0.05 vs. DEP-treated group. AMs were pretreated overnight with TF-343 (100 or 200 *μ*g/ml) before adding DEP (100 *μ*g/ml). At 3 h after DEP stimulation, DCF-DA staining and the MTT assay were performed.

Exposure to DEP or PM induces cell damage via oxidative stress mediated by ROS. We evaluated oxidative stress by measuring the fluorescence intensity of DCF-DA. DEP induced an increase in ROS levels in AMs as compared to vehicle treatment; however, pretreatment with TF-343 reversed this effect (see Figure 5(a)). In addition, viability of AMs was reduced by DEP exposure relative to vehicle-treated cells, as determined with the MTT assay (see Figure 5(b)), but this was alleviated by TF-343 pretreatment.

### 3.4. TF-343 Suppresses Inflammatory Gene Expression in DEP-Stimulated AMs

**Figure 6:**
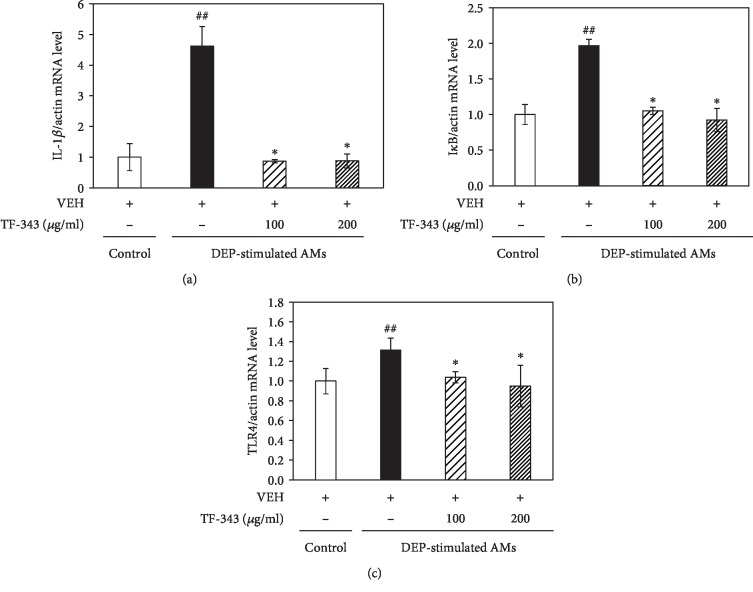
Expression levels of inflammation-related genes including (a) IL-1*β*, (b) I*κ*B, and (c) TLR4 in DEP-stimulated AMs. AMs were pretreated overnight with TF-343 (100 or 200 *μ*g/ml) before adding DEP (100 *μ*g/ml). Real-time PCR analysis was performed 48 h after DEP stimulation. Data represent mean ± SD (*n* = 5 per group). ^##^*P* < 0.01 vs. control, ^∗^*P* < 0.05 vs. DEP-treated group.

We investigated whether TF-343 reduces inflammatory gene expression in DEP-stimulated AMs by real-time PCR analysis and found that inflammatory genes including IL-1*β*, I*κ*B, and TLR4 were upregulated following DEP exposure as compared to vehicle treatment. This effect was abrogated by TF-343 pretreatment (see Figure 6).

### 3.5. TF-343 Prevents I*κ*B Degradation and NF-*κ*B Nuclear Translocation in DEP-Stimulated AMs

**Figure 7:**
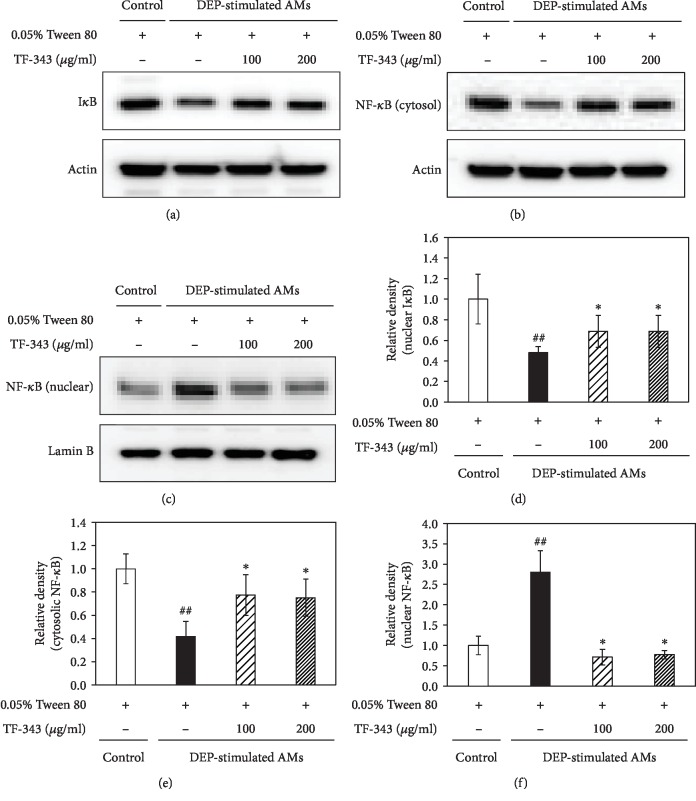
(a–c) Representative western blots and (d–f) relative density of I*κ*B and NF-*κ*B in DEP-stimulated AMs. Data represent the mean ± SD (*n* = 5 per group). ^##^*P* < 0.01 vs. control, ^∗^*P* < 0.05 vs. DEP-treated group.

NF-*κ*B signaling plays an important role in the regulation of inflammatory responses [16]. We investigated whether TF-343 inhibits NF-*κ*B activation in DEP-stimulated AMs. The western blot analysis showed that the I*κ*B level was downregulated whereas nuclear NF-*κ*B p65 accumulation was increased in DEP-stimulated AMs as compared to the vehicle treatment group. TF-343 pretreatment abolished the degradation of I*κ*B and nuclear NF-*κ*B p65 localization induced by DEP (see Figure 7).

## 4. Discussion

The results of this study provide the first demonstration of the protective effect of TF-343, a natural mixture of oriental herbs, in an animal model of DEP-induced lung inflammation. Moreover, we found that TF-343 exerted protective effects in DEP-stimulated AMs by attenuating oxidative stress-associated lung inflammation via modulation of NF-*κ*B activation and IL-1*β* production.

DEP is a major component of air pollutants that has adverse effects on the respiratory and cardiovascular systems [1–9]. DEP components vary depending on the season, region, and source [35]; they can penetrate lung tissue and cause local damage in chronic obstructive pulmonary disease (COPD), asthma, and fibrosis by increasing cell permeability, ROS production, and cytokine secretion [1, 2, 6, 7, 36]. Chronic DEP exposure can lead to DNA damage and mutation, which can subsequently develop into cancer [8, 9].

DEP induces lung inflammation via induction of oxidative stress. ROS are products of normal cellular metabolism and under normal conditions play an important role in the maintaining cellular redox homeostasis [36]. However, overproduction of ROS can lead to lung damage through activation of various signaling pathways such as receptor tyrosine kinase, mitogen-activated protein kinase, NF-*κ*B, and signal transducer and activator of transcription-1 [1, 37]. Activated immune cells including neutrophils and macrophages are a potential source of ROS that stimulate the release of cytokines, lipid mediators, and toxic proteases and cause oxidative stress [38, 39]. Studies in humans have shown that DEP induces the recruitment and activation of inflammatory cells such as macrophages, neutrophils, B and T lymphocytes, and mast cells in the lungs, thereby increasing ROS production [40–42]. We also observed an association between DEP-induced inflammation and ROS. Given the role of ROS as second messengers in many inflammation-related signaling pathways, antioxidants have long been considered as effective therapeutics for the treatment of lung inflammation [43, 44].

TF-343 is an extract of eight natural herbs used in Korean traditional medicine that have well-known nutritional and medicinal effects. *L. japonica* has antibacterial, antioxidative, and antiallergic and immunoregulatory effects [15]. *A. triphylla* var. Japonica has anti-inflammatory and antiobesity and hepatoprotective effects [16–18]. *T. platycarpum* has been used for the treatment of liver disease, anemia, and nervousness and for detoxification [19–21]. *S. chinensis* has anti-inflammatory and antidiabetic activities [22]. *U. davidiana* var. japonica is effective in the treatment of edema and enteritis [23, 24]. *C. tora* has anti-inflammatory and analgesic activities [25]. *G. max* Merrill has demonstrated health-promoting properties including the lowering of cholesterol by blocking its absorption, prevention of human immunodeficiency virus infection, and anticarcinogenic, antihepatotoxic, antimutagenic, and antimurogenic and immunostimulatory activities [26–30]. *G. uralensis* has been used as a raw material for the treatment of hyperlipidemia, atherosclerosis, viral diseases, and allergic inflammation including chronic hepatitis and atopic dermatitis [31, 32]. However, the pharmacologic effects of TF-343 on DEP-induced lung inflammation have not been previously reported.

Firstly, we assessed the potential effect of TF-343 in an animal model of DEP-induced lung inflammation. Our results showed that DEP increased infiltration of inflammatory cells in the lung and recruited black-pigmented AMs in the alveoli of an in vivo model. Importantly, TF-343 pretreatment reduced DEP-induced lung inflammation and infiltration of these AMs in the lungs. These results indicate that TF-343 can protect the lungs against the toxic effects of DEP.

In vitro models have been used to examine the molecular basis for DEP toxicity including DNA damage as well as effects on cell proliferation and differentiation and cytokine and chemokine release [36]. In this study, we investigated the mechanism underlying the protective effects of TF-343 against DEP-induced lung inflammation using cultured AMs; these resident immune cells in the distal lung are a first line of defense to inhaled pathogens and environmental particulates including DEP [45]. AMs maintain air spaces in the respiratory system through phagocytosis of pathogens and debris and infiltrate into regions of tissue damage and release mediators that prime immune responses in the lung such as IL-1, IL-6, and tumor necrosis factor- (TNF-) *α* upon exposure to PM [46–48]. IL-1 family cytokines such as IL-1*α* and IL-1*β* are involved in various aspects of the immune and inflammatory responses and contribute to lung diseases caused by DEP exposure [49]. Acute exposure to PM resulted in increased levels of IL-1 in humans [50], and COPD patients have higher levels of inflammatory cytokines such as IL-6, TNF-*α*, and IL-1*β* and are more susceptible to PM [51].

We investigated the effect of TF-343 on cell viability and the levels of oxidative stress and inflammatory genes in DEP-stimulated AMs and found that viability was decreased after DEP exposure whereas the expression of the proinflammatory cytokine IL-1*β* and oxidative stress was increased. TF-343 pretreatment alleviated these effects.

NF-*κ*B, a multiprotein complex, is involved in the early cellular defense reactions in higher organisms and plays a critical role in many cellular processes including cell proliferation, apoptosis, immune response to infection, and inflammation. Also, the dysregulation of NF-*κ*B signaling is implicated in inflammation-related diseases and cancer [14]. Oxidant/antioxidant imbalance in the lung leads to the activation of this redox-sensitive transcription factor, NF-*κ*B [52]. ROS have also been directly implicated as second messengers in the activation of NF-*κ*B, based on the ability to activate NF-*κ*B by oxidation of a cysteine-SH group or by ubiquitination and proteolysis of I*κ*B [53]. I*κ*B disrupts the dimerization of NF-*κ*B p50/p65, leading to the nuclear translocation of the p65 subunit and the transcription of genes encoding inflammation-related molecules including IL-1*β* [14]; a reduction in the expression of I*κ*B is associated with decreased transcriptional activity of NF-*κ*B. In our study, I*κ*B mRNA expression, which was upregulated by DEP treatment, was decreased by TF-343 pretreatment. Moreover, consistent with these observations, our results showed that NF-*κ*B levels in nuclear protein extracts from AMs are substantially increased in DEP-stimulated AMs. In addition, the increases of proinflammatory mediator, IL-1*β*, and oxidative stress occurred in our DEP-stimulated AMs. TF-343 pretreatment decreased the DEP-induced translocation of NF-*κ*B into the nucleus and increases IL-1*β* and ROS. Thus, our results indicate that TF-343 suppresses NF-*κ*B-mediated gene transcription by suppressing I*κ*B expression in DEP-stimulated AMs, resulting in downregulation of IL-1*β*. NF-*κ*B is a major regulator of proinflammatory gene expression, with nearly 400 target genes encoding cytokines, chemokines, cell adhesion molecules, growth factors, oncogenes, and pro-/antiapoptotic proteins [54, 55]. The results presented here indicate that TF-343 reverses DEP-induced oxidative stress, cytotoxicity, and inflammation via modulation of the NF-*κ*B pathway.

TLR4 recognizes pathogen-associated molecules and initiates the innate immune response, while also regulating the adaptive immune response to PM. Environmental exposure to PM increases TLR4 expression due to the small amounts of microbial materials such as lipopolysaccharide/endotoxin, beta-glucan, bacteria, and fungal spores contained therein [48]. TLR4 activates NF-*κ*B to induce the expression of genes encoding inflammatory mediators such as IL-1*β* [56, 57]. Our results showed that the TLR4 mRNA level was increased in DEP-stimulated AMs, an effect that was abolished by TF-343 pretreatment. Thus, TF-343 may reduce inflammation in part by reversing DEP-induced TLR4 expression in AMs.

In conclusion, our in vitro and in vivo findings indicate that TF-343 has a protective effect against DEP-induced lung inflammation via modulation of NF-*κ*B signaling. Thus, TF-343 may be an effective prophylactic or therapeutic agent for minimizing the risk of DEP-induced lung disorders.

## 5. Conclusions

Our results indicate that TF-343 has a protective effect against DEP-induced lung inflammation. Moreover, TF-343 significantly decreased the activation of NF-*κ*B increased by DEP inhalation. These results suggest that TF-343 may be an effective prophylactic or therapeutic agent for minimizing the risk of DEP-induced lung disorders.

## Figures and Tables

**Table 1:** Quantitative histopathological assessment of lung sections.

Group		Vehicle control	DEP VEH	DEP TF-343 (200)	DEP TF-343 (400)	DEP TF-343 (800)	DEP Dexa (1)
Accumulation of black particle-laden alveolar macrophages and black particles in the alveolar lumen	Minimal	0	0	0	0	3	4
	Mild	0	3	1	4	2	1
	Moderate	0	2	3	1	0	0
	Marked	0	0	1	0	0	0
	Mean ± SD	0	2.4 ± 0.55^##^	3.0 ± 0.71	2.2 ± 0.45	1.4 ± 0.55	1.2 ± 0.45^∗^
Inflammatory cell infiltration	Minimal	0	3	3	2	2	0
	Mild	0	1	0	1	0	0
	Mean ± SD	0	1.0 ± 0.71^#^	0.6 ± 0.55	0.8 ± 0.83	0.4 ± 0.55	0^∗^

1: minimal; 2: mild; 3: moderate; 4: marked. Data are presented as mean ± SD from 5 mice per group. DEP: diesel exhaust particulate; Dexa: dexamethasone; VEH: vehicle. ^#^*P* < 0.05 vs. vehicle control, ^##^*P* < 0.01 vs. vehicle control, ^∗^*P* < 0.05 vs. DEP-treated group.

## Data Availability

The data used to support the findings of this study are included within the article.

## References

[1] Rodriguez C. I. F., Vargas A. R. O., Ovalle I. S., Medina P. S. (2016). Aeroparticles, composition, and lung diseases. *Frontiers in Immunology*.

[2] Löndahl J., Swietlicki E., Rissler J. (2012). Experimental determination of the respiratory tract deposition of diesel combustion particles in patients with chronic obstructive pulmonary disease. *Particle and Fibre Toxicology*.

[3] Ito Y., Yanagiba Y., Ramdhan D. H. (2016). Nanoparticle-rich diesel exhaust-induced liver damage via inhibited transactivation of peroxisome proliferator-activated receptor alpha. *Environmental Toxicology*.

[4] Chan T. C., Zhang Z., Lin B. C. (2018). Long-term exposure to ambient fine particulate matter and chronic kidney disease: a cohort study. *Environmental Health Perspectives*.

[5] Bates J. T., Weber R. J., Abrams J. (2015). Reactive oxygen species generation linked to sources of atmospheric particulate matter and cardiorespiratory effects. *Environmental Science & Technology*.

[6] Darquenne C. (2014). Aerosol deposition in the human lung in reduced gravity. *Journal of Aerosol Medicine and Pulmonary Drug Delivery*.

[7] Nemmar A., Holme J. A., Rosas I., Schwarze P. E., Alfaro-Moreno E. (2013). Recent advances in particulate matter and nanoparticle toxicology: a review of the in vivo and in vitro studies. *BioMed Research International*.

[8] Atkinson R. W., Kang S., Anderson H. R., Mills I. C., Walton H. A. (2014). Epidemiological time series studies of PM2.5 and daily mortality and hospital admissions: a systematic review and meta-analysis. *Thorax*.

[9] Cai Y., Zhang B., Ke W. (2016). Associations of short-term and long-term exposure to ambient air pollutants with hypertension: a systematic review and meta-analysis. *Hypertension*.

[10] Li N., Wang M., Oberley T. D., Sempf J. M., Nel A. E. (2002). Comparison of the pro-oxidative and proinflammatory effects of organic diesel exhaust particle chemicals in bronchial epithelial cells and macrophages. *The Journal of Immunology*.

[11] Li N., Venkatesan M. I., Miguel A. (2000). Induction of heme oxygenase-1 expression in macrophages by diesel exhaust particle chemicals and quinones via the antioxidant-responsive element. *The Journal of Immunology*.

[12] International Agency for Research on Cancer (2013). *Outdoor Air Pollution a Leading Environmental Cause of Cancer Deaths*.

[13] Arulselvan P., Fard M. T., Tan W. S. (2016). Role of antioxidants and natural products in inflammation. *Oxidative Medicine and Cellular Longevity*.

[14] Miller S. C., Huang R., Sakamuru S. (2010). Identification of known drugs that act as inhibitors of NF-*κ*B signaling and their mechanism of action. *Biochemical Pharmacology*.

[15] Che H., Ha D., Wei Y., Zheng S. (2012). Characterization and anti-allergic effect of a polysaccharide from the flower buds of Lonicera japonica. *Carbohydrate Polymers*.

[16] Yoon Y. P., Lee H. J., Lee D. U., Lee S. K., Hong J. H., Lee C. J. (2015). Effects of lupenone, lupeol, and taraxerol derived from Adenophora triphylla on the gene expression and production of airway MUC5AC mucin. *Tuberculosis and Respiratory Diseases*.

[17] Kim S. J., Cho H. I., Kim S. J. (2014). Protective effects of lupeol against D-galactosamine and lipopolysaccharide-induced fulminant hepatic failure in mice. *Journal of Natural Products*.

[18] Lee D. R., Lee Y. S., Choi B. K. (2015). Roots extracts of Adenophora triphylla var. japonica improve obesity in 3T3-L1 adipocytes and high-fat diet-induced obese mice. *Asian Pacific Journal of Tropical Medicine*.

[19] Park C. M., Jin K. S., Lee Y. W., Song Y. S. (2011). Luteolin and chicoric acid synergistically inhibited inflammatory responses via inactivation of PI3K-Akt pathway and impairment of NF-*κ*B translocation in LPS stimulated RAW 264.7 cells. *European Journal of Pharmacology*.

[20] Clare B. A., Conroy R. S., Spelman K. (2009). The diuretic effect in human subjects of an extract of Taraxacum officinale folium over a single day. *Journal of Alternative and Complementary Medicine*.

[21] Onal S., Timur S., Okutucu B., Zihnioğlu F. (2005). Inhibition of *α*-glucosidase by aqueous extracts of some potent antidiabetic medicinal herbs. *Preparative Biochemistry & Biotechnology*.

[22] Yu M. H., Im H. G., Lee J. W. (2008). Effects of ethanol extract from Saururus chinensis (Bour.) Baill on lipid and antioxidant metabolisms in rats fed a high-fat diet. *Natural Product Research*.

[23] Yang W. K., Lee J. J., Sung Y. Y., Kim D. S., Myung C. S., Kim H. K. (2013). Extract of Ulmus macrocarpa Hance prevents thrombus formation through antiplatelet activity. *Molecular Medicine Reports*.

[24] Lee M. Y., Seo C. S., Ha H. (2010). Protective effects of Ulmus davidiana var. japonica against OVA-induced murine asthma model via upregulation of heme oxygenase-1. *Journal of Ethnopharmacology*.

[25] Antonisamy P., Dhanasekaran M., Kim H. R., Jo S. G., Agastian P., Kwon K. B. (2017). Anti-inflammatory and analgesic activity of ononitol monohydrate isolated from Cassia tora L. in animal models. *Saudi Journal of Biological Sciences*.

[26] Zang Y., Hashimoto S., Yu C., Igarashi K. (2018). Protective effects of dietary kaempferol glycoside components from unripe soybean (edamame, Glycine max L. Merrill. ‘Jindai’) leaves and their serous metabolite on carbon tetrachloride-induced liver injury mice. *Journal of Food Science and Technology*.

[27] Kinjo J., Imagire M., Udayama M., Arao T., Nohara T. (1998). Structure-hepatoprotective relationship of soyasaponins I–IV having soyasapogenogenol B as aglycone. *Planta Medica*.

[28] Miyao H., Arao T., Udayama M., Kijo J., Nohara T. (1998). Kaikasaponin III and soyasaponin I, major triterpene saponins of abrus cantoneiensis act on GOT and GPT: influence of transamoase elevation of rat liver cells concomitantly exposed to CCl_4_ for one hour. *Planta Medica*.

[29] Hu J., Reddy M. B., Hendrich S., Murphy P. A. (2004). Soyasaponin I and sapogenol B have limited absorption by Caco-2 intestinal cells and limited bioavailability in women. *The Journal of Nutrition*.

[30] Berhow M. A., Wagner E. D., Vaughn S. F., Plewa M. J. (2000). Characterization and antimutagenic activity of soybean saponins. *Mutation Research/Fundamental and Molecular Mechanisms of Mutagenesis*.

[31] Yang N., Patil S., Zhuge J. (2013). Glycyrrhiza uralensis flavonoids present in anti-asthma formula, ASHMI™, inhibit memory Th2 responses in vitro and in vivo. *Phytotherapy Research*.

[32] Jung J. C., Lee Y. H., Kim S. H. (2016). Hepatoprotective effect of licorice, the root of Glycyrrhiza uralensis Fischer, in alcohol-induced fatty liver disease. *BMC Complementary and Alternative Medicine*.

[33] Provoost S., Maes T., Willart M. A., Joos G. F., Lambrecht B. N., Tournoy K. G. (2010). Diesel exhaust particles stimulate adaptive immunity by acting on pulmonary dendritic cells. *The Journal of Immunology*.

[34] Renne R., Brix A., Harkema J. (2009). Proliferative and non-proliferative lesions of the rat and mouse respiratory tract. *Toxicologic Pathology*.

[35] Jia Y. Y., Wang Q., Liu T. (2017). Toxicity research of PM2.5 compositions in vitro. *International Journal of Environmental Research and Public Health*.

[36] Schwarze P. E., Totlandsdal A. I., Låg M., Refsnes M., Holme J. A., Øvrevik J. (2013). Inflammation-related effects of diesel engine exhaust particles: studies on lung cells in vitro. *BioMed Research International*.

[37] Byrne J. D., Baugh J. A. (2008). The significance of nanoparticles in particle-induced pulmonary fibrosis. *McGill Journal of Medicine*.

[38] Emmendoerffer A., Hecht M., Boeker T., Mueller M., Heinrich U. (2000). Role of inflammation in chemical-induced lung cancer. *Toxicology Letters*.

[39] Bartsch H., Nair J. (2006). Chronic inflammation and oxidative stress in the genesis and perpetuation of cancer: role of lipid peroxidation, DNA damage, and repair. *Langenbeck's Archives of Surgery*.

[40] Frampton M. W. (2001). Systemic and cardiovascular effects of airway injury and inflammation: ultrafine particle exposure in humans. *Environmental Health Perspectives*.

[41] Pourazar J., Mudway I. S., Samet J. M. (2005). Diesel exhaust activates redox-sensitive transcription factors and kinases in human airways. *American Journal of Physiology-Lung Cellular and Molecular Physiology*.

[42] Snow S. J., De Vizcaya-Ruiz A., Osornio-Vargas A. (2014). The effect of composition, size, and solubility on acute pulmonary injury in rats following exposure to Mexico city ambient particulate matter samples. *Journal of Toxicology and Environmental Health, Part A*.

[43] Sauer H., Wartenberg M., Hescheler J. (2001). Reactive oxygen species as intracellular messengers during cell growth and differentiation. *Cellular Physiology and Biochemistry*.

[44] Mittal M., Siddiqui M. R., Tran K., Reddy S. P., Malik A. B. (2014). Reactive oxygen species in inflammation and tissue injury. *Antioxidants & Redox Signaling*.

[45] Morales-Nebreda L., Misharin A. V., Perlman H., Budinger G. R. (2015). The heterogeneity of lung macrophages in the susceptibility to disease. *European Respiratory Review*.

[46] Renwick L. C., Donaldson K., Clouter A. (2001). Impairment of alveolar macrophage phagocytosis by ultrafine particles. *Toxicology and Applied Pharmacology*.

[47] Lawal A. O. (2018). Diesel exhaust particles and the induction of macrophage activation and dysfunction. *Inflammation*.

[48] Hiraiwa K., van Eeden S. F. (2013). Contribution of lung macrophages to the inflammatory responses induced by exposure to air pollutants. *Mediators of Inflammation*.

[49] Yang H. M., Ma J. Y., Castranova V., Ma J. K. (1997). Effects of diesel exhaust particles on the release of interleukin-1 and tumor necrosis factor-alpha from rat alveolar macrophages. *Experimental Lung Research*.

[50] Amakawa K., Terashima T., Matsuzaki T., Matsumaru A., Sagai M., Yamaguchi K. (2003). Suppressive effects of diesel exhaust particles on cytokine release from human and murine alveolar macrophages. *Experimental Lung Research*.

[51] Sunyer J., Schwartz J., Tobías A., Macfarlane D., Garcia J., Antó J. M. (2000). Patients with chronic obstructive pulmonary disease are at increased risk of death associated with urban particle air pollution: a case-crossover analysis. *American Journal of Epidemiology*.

[52] Jr Henderson W. R., Chi E. Y., Teo J. L., Nguyen C., Kahn M. (2002). A small molecule inhibitor of redox-regulated NF-*κ*B and activator protein-1 transcription blocks allergic airway inflammation in a mouse asthma model. *Journal of Immunology*.

[53] Rahman I., MacNee W. (1998). Role of transcription factors in inflammatory lung diseases. *Thorax*.

[54] Ahn K. S., Aggarwal B. B. (2005). Transcription factor NF-*κ*B: a sensor for smoke and stress signals. *Annals of the New York Academy of Sciences*.

[55] Lee C. H., Jeon Y. T., Kim S. H., Song Y. S. (2007). NF-*κ*B as a potential molecular target for cancer therapy. *BioFactors*.

[56] Kawai T., Akira S. (2010). The role of pattern-recognition receptors in innate immunity: update on Toll-like receptors. *Nature Immunology*.

[57] Georas S. N., Upham J. (2014). Environmental exposures and innate immunity in the lung. *Journal of Enviromental Immunology and Toxicology*.

